# Seroprevalence of cytomegalovirus IgG and IgM antibodies and associated factors among women with spontaneous abortion in Lahj Governorate, Yemen

**DOI:** 10.1186/s13104-026-07983-4

**Published:** 2026-08-15

**Authors:** Ali N. M. Gubran, Marwa Mohammed S. Masood, Alaa Hatem Othman Qasim, Eglal Yones Abdulwali Ali, Eman Abdulaziz Taha Alattas, Farah Maher A. F. Khan, Hafidah Saleh A. Saleh, Samah Nabeel A. Mohammed, Shahd Abd Almonem A. Saleh, Shaima Mohammed A. Othman, Shams Mohammed A. Saleh, Sufyan Hassan A. Nasser, Wafa Yaslam O. Al-Sharafi

**Affiliations:** 1 Department of Health Sciences, Faculty of Medicine and Health Sciences, University of Science and Technology, Aden, Yemen; 2 Medical Laboratory Department, Faculty of Medicine and Health Sciences, University of Al-Reyada, Aden, Yemen

**Keywords:** Cytomegalovirus, Seroprevalence, IgG, IgM, Spontaneous abortion, Lahj, Yemen

## Abstract

**Objectives:**

This study aimed to determine the seroprevalence of anti-CMV IgG and anti-CMV IgM antibodies among women with spontaneous abortion in Lahj Governorate, Yemen. An analytical cross-sectional study was among 110 women with spontaneous abortion. Five milliliters of blood were collected from women, sera were separated, and anti-CMV IgG and IgM were determined using a Cobas^®^ e 411 analyzer that is based on electrochemiluminescence immunoassay (ECLIA). The data were entered and then analyzed using SPSS. Associations were assessed using the chi-square test, and prevalence ratios (PR) with 95% confidence intervals (CIs) were calculated.

**Results:**

The seroprevalence of anti-CMV IgG and IgM antibodies was 80.9% and 7.3%, respectively. A statistically significant association was observed between anti-CMV IgM seropositivity and having experienced two previous spontaneous abortions (PR = 5.0, 95% CI: 1.13–21.97; *p* = 0.042). A high seroprevalence of CMV IgG antibodies and low IgM seropositivity among women with spontaneous abortion in Lahj, Yemen.

## Introduction

The cytomegalovirus (CMV) infection is a herpesviridae member that is the most common cause of miscarriage (abortion) among pregnant women [1, 2]. It is the leading cause of stillbirth and congenital abnormalities among fetuses and newborns, respectively [3]. Its infection may occur as primary, recurrent, or reactivation of latent infection [4]. The severity of infection increased in pregnant women, immunocompromised patients, organ transplant recipients, those on immunosuppressive therapy, and AIDS patients [5, 6].

The virus can be shed in most body fluids and secretions, including saliva, tears, breast milk, and genital tract secretions [7, 8]. The fetus can acquire a CMV infection from an infected mother through the placenta [9] and the newborn through breastfeeding and during delivery (birth canal) [10]. Sexual contact, kissing, unscreened transfusion of contaminated blood or blood products, and organ transplants are the common routes of transmission between adults [11, 12].

Globally, the prevalence of CMV antibodies among women with spontaneous abortion varies, where immunoglobulin G (IgG) antibodies ranged from 4.1% to 100% versus 0% to 93% immunoglobulin M (IgM) antibodies [13–16].

In Yemen, the seropositive IgG and IgM against CMV in four different governorates were 77.6% and 83.3% in Aden [2], 99% and 3.8% in Abyan [17], 98.7% and 7.33% in Ibb [18], and 99.3% and 3.7% in Dhamar, respectively [19].

Hoverer, no single report about the seroprevalence of CMV among women with spontaneous abortion in Lahj Governorate. Understanding the magnitude of CMV infections is important for applying the appropriate preventive measures to control the virus, thereby reducing the burden of abortion and congenital disorders. Therefore, this study was conducted for the first time in this governorate and aimed to determine the prevalence of anti-CMV IgG and anti-CMV IgM antibodies among women with spontaneous abortion in Lahj Governorate, Yemen, and to identify factors associated with seropositivity among women with spontaneous abortion.

## Subjects and methods

### Study design and setting

This study was conducted during a period from 1 February to 15 June 2025 in Lahj Governorate, Yemen. Those women were attended at Ibn Khaldun Hospital, which is a private healthcare facility providing obstetric and gynecological services in Lahj Governorate. In this cross-sectional study, exposure variables and serological outcomes were measured simultaneously, without participant follow-up [20].

### Study population

The study population consisted of women of reproductive age with a history of spontaneous abortion who attended the selected health facilities during the study period.

### Inclusion and exclusion criteria

Women who attended to receive healthcare at Ibn Khaldun Hospital in Lahj Governorate due to exposure to spontaneous abortion but were clinically stable without abortion-related complications and those non-pregnant women who had experienced spontaneous abortion within the previous three months agreed to participate and provided written informed consent and were included in the study. Women without a history of spontaneous abortion or with any abortion-related complications; those pregnant women with ongoing pregnancies who were not exposed to abortion; female children and adolescents younger than 18 years; single women; and those reporting immunocompromising conditions based on self-reported medical history (including chronic diseases such as HIV infection, malignancy, or other conditions requiring immunosuppressive therapy) were excluded from the study.

### Sample size

The sample size was estimated using the prevalence of CMV antibodies reported among women with spontaneous abortion in Abyan Governorate, Yemen, where the seroprevalence of CMV IgG and IgM antibodies was 98.67% and 7.33%, respectively [17]. The required sample size was calculated using Stephen Thompson’s formula [21]:

$$\:N=\frac{{Z}^{2}P(1-P)}{{d}^{2}}$$

N = expected minimum sample size; Z = standard, corresponding to 95% confidence (1.96); P = the % estimated prevalence, which was 7.33% for IgM antibodies against CMV but not 98.67% for IgG; and D = the maximum likely error taken as 5%. The calculated sample size was 105 women, and adding 10% non-response, the total sample size was 115.5 (~ 116) women. However, during the data collection period, 110 women were enrolled, yielding a response rate of approximately 94.8%, which is considered acceptable for studies involving women with spontaneous abortion. The calculated sample size came from CMV IgM prevalence (7.33%), while the CMV IgG prevalence (98.67%) was not used for the calculation of sample size because it resulted in a minimum estimated sample size of 21 women, which was insufficient for obtaining a reliable CMV prevalence rate and risk factor associations.

### Data collection

Data were collected through face-to-face interviews using a structured questionnaire after obtaining written informed consent from all participants. The questionnaire was adapted from previously published studies and was modified to meet the objectives of the present study [2, 17, 18]. It included sociodemographic variables such as age, educational level, residence, occupation, and economic status. Economic status was classified into three categories (low, middle, and high) based on self-reported monthly household income using predefined criteria included in the study questionnaire. The income thresholds were determined based on the local socioeconomic context in Yemen. The questionnaire also included obstetric and clinical characteristics, including history of primary abortion, recurrent spontaneous abortion, number of abortions, trimester of abortion among women with recurrent spontaneous abortion, duration of marriage, number of pregnancies, and number of live births. The presence of symptoms related to infection among women were noted, and two commonly important risk factors include exposing women to blood transfusion and use of corticosteroid drugs [2, 17, 18].

Recurrent spontaneous abortion was defined as the occurrence of two or more consecutive spontaneous pregnancy losses before fetal viability [22]. Women with known immunocompromising conditions were identified based on a structured questionnaire item on medical history, including self-reported diagnoses of chronic diseases that impaired immune function, such as HIV infection, malignancies, autoimmune diseases, organ transplantation, and use of immunosuppressive therapy.

### Laboratory procedures

Approximately 5 milliliters of venous blood were collected aseptically from each participant into plain vacutainer tubes. Samples were transported to the laboratory of Tour Sina Hospital, Saber City, Lahj Governorate. After clotting, serum was separated by centrifugation at 3000 rpm for 10 min and stored at − 80 °C until laboratory analysis.

Serological testing for anti-Cytomegalovirus (CMV) IgG and IgM antibodies was performed using the Elecsys^®^ CMV IgG and Elecsys^®^ CMV IgM electrochemiluminescence immunoassays (ECLIA) on the cobas^®^ e 411 analyzer (Roche Diagnostics GmbH, Mannheim, Germany), according to the manufacturer’s instructions. The ECLIA technique is a fully automated immunoassay based on electrochemiluminescent detection, widely used for high-sensitivity serological testing of infectious diseases, including CMV [23–26].

The assay is based on a double-antigen sandwich principle for IgG detection and a capture immunoassay principle for IgM detection. In brief, patient serum is incubated with recombinant CMV antigens labeled with biotin and ruthenium complexes to form antigen–antibody complexes. Streptavidin-coated microparticles are added, and the complexes are magnetically captured onto an electrode surface. Application of voltage induces chemiluminescent emission, which is measured by a photomultiplier. The emitted signal is proportional to the antibody concentration in the sample [25, 26]. Calibration of the cobas^®^ e 411 analyzer was performed using Roche calibrators prior to testing, and internal quality-control materials provided by the manufacturer were analyzed with each run to ensure analytical accuracy and precision. Results were automatically interpreted by the system according to manufacturer-defined cut-off values and reported as negative, equivocal, or positive. Values above the upper cutoff were considered positive; below the lower cutoff, negative; and those within the grey zone, equivocal, as defined in the assay instructions [24]. All reagents, calibrators, and control materials were supplied by Roche Diagnostics GmbH, Mannheim, Germany.

### Data analysis

The data were entered and analyzed using the Statistical Package for the Social Sciences (SPSS^®^) software (Version 26). Almost all data were qualitative and were represented as numbers and percentages. Univariate analysis examined associations between CMV seropositivity and exposure variables using chi-square (or Fisher’s exact when expected cell count < 5). Prevalence Ratios (PR) and 95% confidence intervals (CIs) were calculated to estimate the strength of associations. PRs were calculated as the ratio of the prevalence of CMV seropositivity among exposed women divided by the prevalence among unexposed women (PR = prevalence in exposed / prevalence in unexposed). The 95% CIs for PRs were calculated using the standard error of the natural logarithm of the PR. This approach is more appropriate for cross-sectional studies than odds ratios, as it directly measures prevalence rather than approximating risk or odds [27]. Multivariable analysis was not performed due to limited sample size, particularly for IgM-positive cases (*n* = 8), which precluded stable multivariable modeling.

## Results

The women with spontaneous abortion (*n* = 110) included in this study had a median age of 26 (IQR = 7) years. According to the sociodemographic characteristics of women, the majority of women were 36 (32.7%) in the age group 26–29 years, 61 (55.5%) had a secondary school education level, 76 (69.1%) were from urban areas, 72 (65.5%) had a medium income, and 74 (67.3%) were housewives (Table 1).

**Table 1:** The sociodemographic characteristics of 110 women with spontaneous abortion in Lahj, Yemen

Variable	Number	Percentage (%)
Age (years)		
< 22	30	27.3
22–25	23	20.9
26–29	36	32.7
> 29	21	19.1
Education level		
Illiterate	7	6.4
Primary school	22	20.0
Secondary school	61	55.5
University	20	18.2
Residence		
Rural	34	30.9
Urban	76	69.1
Socioeconomic status (income)		
Low	25	22.7
Medium	72	65.5
High	13	11.8
Occupation		
Housewife	74	67.3
Daily work	8	7.3
Private work	18	16.4
Employment	10	9.1

**Fig. 1:**
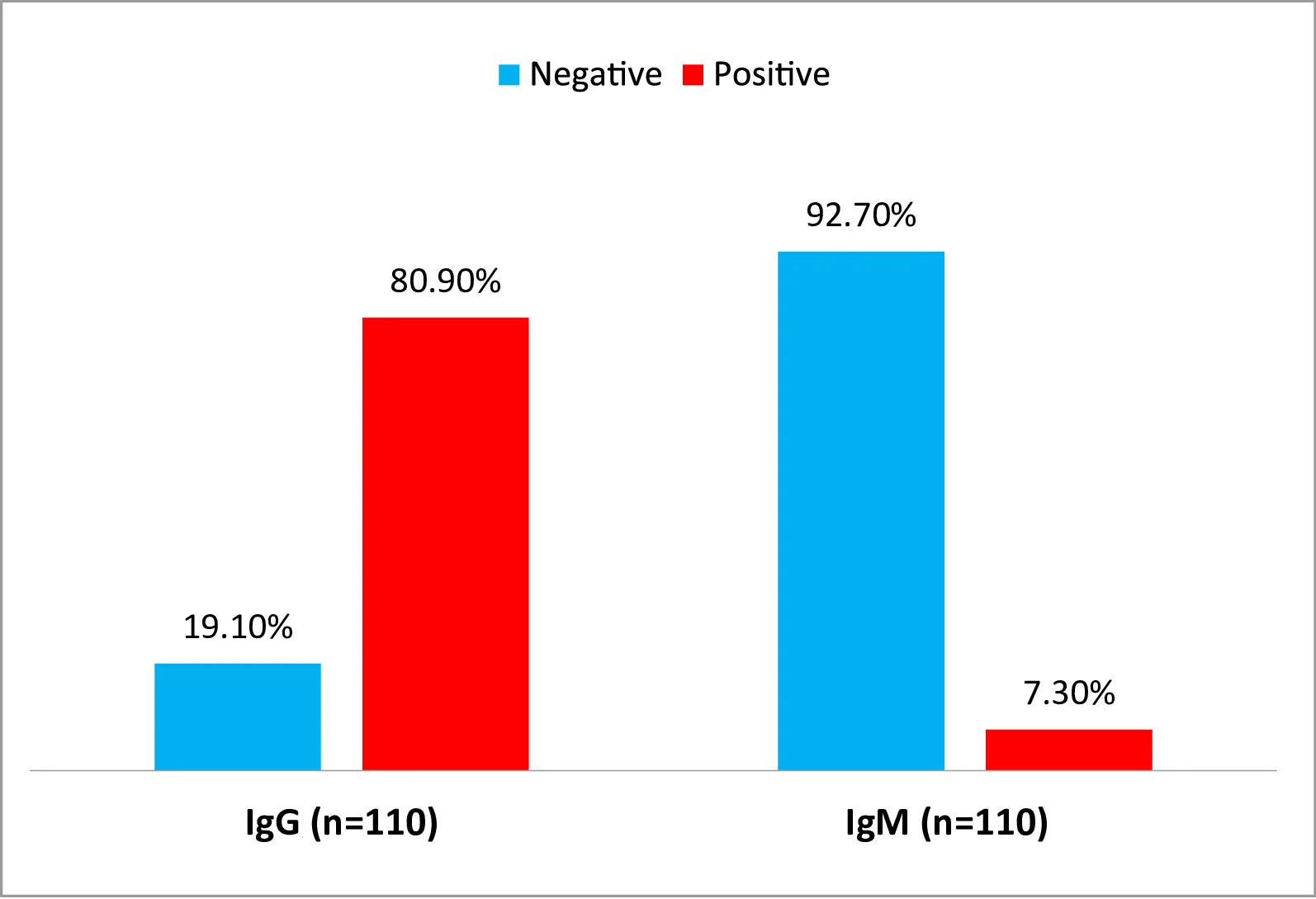
Prevalence of CMV serological markers IgG and IgM women with spontaneous abortion in Lahj, Yemen

The overall prevalence of IgG and IgM antibodies to CMV infections was 89 (80.9%) and 8 (7.3%), respectively (Fig. 1).

Regarding the sociodemographic status, the highest IgG seropositivity was 87.0% among women aged 22–25 years, 85.2% of those with secondary education, 84.2% of urban residents, 100% of those with a high income, and 100% of daily workers. Only low-income women showed a borderline significant association with positive IgG rates (*p* = 0.06). Conversely, the prevalence of IgM was high (11.1%) among women aged 26–29 years, (14.3%) illiterate women, (17.6%) rural women, (15.4%) those with high income, and (9.5%) housewives. No statistically significant associations were observed (Table 2).

**Table 2:** Prevalence of CMV serological markers IgG and IgM among women with spontaneous abortion related to their sociodemographic status in Lahj, Yemen

Category	IgG positive *n*(%)	PR (95% CI)	*P*-value	IgM positive *n*(%)	PR (95% CI)	*P*-value
Age groups/years						
< 22 (*n* = 30)	23 (76.7)	1.43 (0.52–3.99)	0.330	3 (10.0)	1.66 (0.37–7.45)	0.378*
22–25 (*n* = 23)	20 (87.0)	0.58 (0.15–2.15)	0.308	1 (4.3)	UD	UD
26–29 (*n* = 36)	29 (80.6)	1.04 (0.38–2.84)	0.569	4 (11.1)	2.18 (0.51–9.30)	0.240*
> 29 (*n* = 21)	17 (81.0)	0.98 (0.30–3.34)	0.632	0 (0.0)	UD	UD
Educational level						
Illiterate (*n* = 7)	4 (57.1)	0.28 (0.06–1.37)	0.126*	1 (14.3)	UD	UD
Primary (*n* = 22)	18 (81.8)	1.07 (0.32–3.59)	0.586	2 (9.1)	1.36 (0.26–7.28)	0.502*
Secondary (*n* = 61)	52 (85.2)	1.87 (0.72–4.90)	0.148	4 (6.6)	0.79 (0.19–3.33)	0.514*
University (*n* = 20)	15 (75.0)	0.65 (0.21–2.04)	0.322	1 (5.0)	UD	UD
Residence						
Rural (*n* = 34)	25 (73.5)	0.51 (0.20–1.38)	0.146	6 (17.6)	1.37 (0.21–7.17)	0.527*
Urban (*n* = 76)	64 (84.2)			2 (2.6)		
Economic status						
Low (*n* = 25)	17 (68.0)	0.38 (0.14–1.10)	0.060	2 (8.0)	1.14 (0.22–6.06)	0.581*
Medium (*n* = 72)	59 (81.9)	1.20 (0.45–3.20)	0.444	4 (5.6)	0.50 (0.12–2.12)	0.278*
High (*n* = 13)	13 (100.0)	UD	UD	2 (15.4)	2.75 (0.49–15.37)	0.240*
Occupation						
Housewife (*n* = 74)	60 (81.1)	1.03 (0.38–2.84)	0.569	7 (9.5)	3.65 (0.43–30.90)	0.195
Daily (*n* = 8)	8 (100.0)	UD	UD	0 (0.0)	UD	UD
Private (*n* = 18)	14 (77.8)	0.79 (0.23–2.71)	0.465	1 (5.6)	UD	UD
Employment (*n* = 10)	7 (70.0)	0.51 (0.12–2.17)	0.291	0 (0.0)	UD	UD

PR: Prevalence Ratios; 95% CI: 95% confidence interval; UD: Undefined

*Fisher’s exact test; *P-value < 0.05 significant

**Fig. 2:**
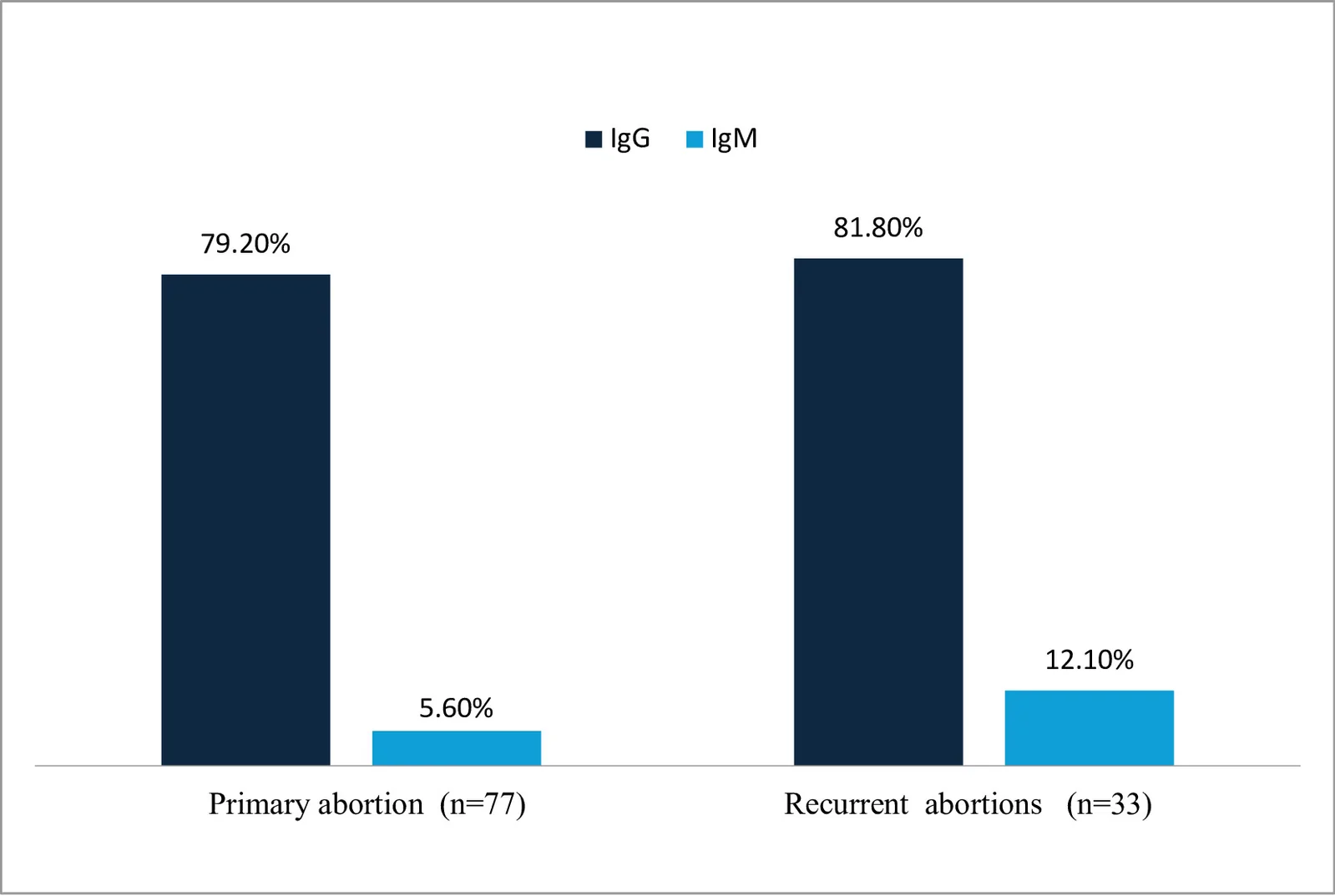
Prevalence of CMV serological markers IgG and IgM according to primary and recurrent abortion among women in Lahj, Yemen

The present data detected that anti-CMV IgG and IgM antibodies among women who had recurrent abortions were 27 (81.8%) and 4 (12.1%), respectively (Fig. 2).

Regarding CMV seroprevalence by obstetric history, the highest anti-CMV IgG and IgM seropositivity rates were (77.8%, 19.0%) in women with three and two previous abortions, (86.1%, 8.2%)) second and first trimesters abortion, (100%, 13%) marriage duration 7–9 years and < 2 years, (100%, 9%) had three and < 3 pregnancies and (83.3%,10.0%) had 1–2 and no live children, respectively. All results were not statistically significant (*p* > 0.05), except a statistically significant association was observed between positive anti-CMV IgM antibodies and women who had experienced two previous abortions (PR = 5.0, 95% CI: 1.13–21.97; *p* = 0.042) (Table 3).

**Table 3:** Prevalence of CMV serological markers IgG and IgM among women with spontaneous abortion according to number of recurrent abortions and trimesters of abortion, period of marriage, number of pregnancies, and live births in Lahj, Yemen

Category	IgG positive *n*(%)	PR (95% CI)	*P*-value	IgM positive *n*(%)	PR (95% CI)	*P*-value
Number of recurrent abortion (*n* = 33)						
Twice (*n* = 21)	16 (76.2)	0.70 (0.22–2.19)	0.368	4 (19.0)	5.00 (1.13–21.97)	0.042*
Third (*n* = 9)	7 (77.8)	0.81 (0.16–4.18)	0.544	0 (0.0)	UD	UD
More than 3 (*n* = 3)	1 (33.3)	UD	UD	0 (0.0)	UD	UD
Trimesters						
1st trimester (*n* = 61)	47 (77.0)	0.56 (0.21–1.51)	0.183	5 (8.2)	1.36 (0.31–6.03)	0.486
2nd trimester (*n* = 36)	31 (86.1)	1.71 (0.57–5.11)	0.242	2 (5.6)	0.66 (0.13–3.48)	0.480
3rd trimester (*n* = 13)	11 (84.6)	1.34 (0.27–6.55)	0.530	1 (7.7)	UD	UD
Period of marriage						
< 2 years (*n* = 23)	16 (69.6)	0.44 (0.15–1.26)	0.107	3 (13.0)	2.46 (0.54–11.16)	0.218*
2–6 years (*n* = 48)	40 (83.3)	1.32 (0.50–3.51)	0.375	3 (6.3)	0.76 (0.17–3.35)	0.508*
7–9 years (*n* = 19)	19 (100.0)	UD	UD	2 (10.5)	1.66 (0.31–8.97)	0.417*
> 9 years (*n* = 20)	14 (70.0)	0.47 (0.16–1.41)	0.145	0 (0.0)	UD	UD
Number of pregnancies						
< 3 (*n* = 89)	72 (80.9)	1.00 (0.30–3.34)	0.632	8 (9.0)	0.91 (0.85–0.97)	0.172
3 (*n* = 11)	11 (100.0)	UD	UD	0 (0.0)	UD	UD
> 3 (*n* = 10)	6 (60.0)	0.31 (0.08–1.20)	0.095	0 (0.0)	UD	UD
Number of live births						
None (*n* = 80)	65 (81.3)	1.08 (0.38–3.11)	0.539	8 (10.0)	0.90 (0.84–0.97)	0.071
1–2 children (*n* = 18)	15 (83.3)	1.21 (0.32–4.65)	0.535	0 (0.0)	UD	UD
> 2 children (*n* = 12)	9 (75.0)	0.68 (0.17–2.74)	0.411	0 (0.0)	UD	UD

PR: Prevalence Ratios; 95% CI: 95% confidence interval; UD: Undefined

*Fisher’s exact test; *P-value < 0.05 significant

Regarding symptoms and risk factors, the highest rates of IgG and IgM antibodies were 81.8% and 18.2% among women with skin rash, 75.0% and 0.0% among those with lymphadenopathies, 88.5% and 9.6% among women who received blood transfusions, and 75.0% and 16.7% among those who used corticosteroids, respectively. These results were not statistically significant (*p* > 0.05) (Table 4).

**Table 4:** Prevalence of CMV serological markers IgG and IgM among women with spontaneous abortion according to symptoms and associated risk factors in Lahj, Yemen

Category	Anti-CMV IgG *n*(%)	PR (95% CI)	*P*-value	Anti-CMV IgM *n*(%)	PR (95% CI)	*P*-value
Symptoms						
Skin rash (*n* = 11)	9 (81.8)	1.06 (0.21–5.35)	0.649	2 (18.2)	3.40 (0.60–19.60)	0.182*
Lymphadenopathy (*n* = 4)	3 (75.0)	0.70 (0.07–7.10)	0.577*	0 (0.0)	UD	UD
Risk factors						
Blood transfusion (*n* = 52)	46 (88.5)	1.19 (0.996–1.430)	0.057	5 (9.6)	2.00 (0.44–8.60)	0.299
Use corticosteroid (*n* = 12)	9 (75.0)	0.68 (0.17–2.74)	0.411	2 (16.7)	3.10 (0.55–17.30)	0.211*

PR: Prevalence Ratios; 95% CI: 95% confidence interval; UD: Undefined

*Fisher’s exact test; P-value < 0.05 significant

## Discussion

This study provided the first documented seroprevalence of anti-CMV IgG antibodies in Lahj governorate, Yemen, which was 80.9%, indicating that most women with spontaneous abortion were previously exposed to CMV. A study from Bangladesh revealed that 82% of women with prior miscarriages had positive anti-CMV IgG [28]. The current finding was lower than that reported by several previous studies conducted among women with spontaneous abortion in Yemen, Iraq, Sudan, Iran, Tanzania, Niger, and India [1, 15–19, 29–36]. On the contrary, this data was higher than other previous studies performed in Yemen, Sudan, Iraq, Libya, Iran, and Leningrad [2, 13, 37–41]. Result variations may be attributed to differences in socio-demographic status, cultures and behaviors of studied groups, and hygiene practices. specimen’s collection time, the sensitivity and specificity of diagnostic CMV techniques, and selected markers [2, 18, 29]. The seroconversion of CMV-specific IgG antibodies takes several weeks after the initial infection or undetectable levels of antibodies [42].

The seropositivity of CMV IgM antibodies in this study was in 8 out of 110 women with spontaneous abortion (7.3%). Similar results were reported in Iraq and Yemen [15, 18], but lower than this finding was shown in Sudan, Libya, Iran, India, and Niger [2, 13, 16, 31, 33, 35, 36, 38, 40–44]. Conversely, various studies conducted in Yemen, Iraq, Sudan, and Iran noticed lower rates than ours [17, 19, 31, 38, 39, 45, 46]. A study also undertaken among Iranian women who had abortions revealed that there were no cases of positive CMV IgM antibodies [47]. Discrepancies may result from differences in timing of maternal and fetal infection and maternal immune status [48]. The timing of serodiagnosis of CMV-specific IgM is also very essential because the IgM antibodies typically appear early after CMV infection and then decline to become undetectable or disappear within a few months [49]. The cross-reactivity of anti-CMV IgM with other herpesviruses, influenza viruses, measles virus, and some autoimmune diseases leads to false positive CMV results [49].

In the present data, four of the CMV IgM-positive women with spontaneous abortion of a total of eight positive cases were exposed to recurrent abortions. It was in agreement with three studies conducted in Niger and Yemen [2, 17, 35]. This data was incompatible with that revealed in Iraq and Sudan [14, 43]. The presence of CMV IgG antibodies may indicate past exposure to the virus, while positive IgM antibodies may indicate a recent acute CMV infection [32]. The primary infection requires additional testing, such as a CMV IgG avidity test and/or polymerase chain reaction (PCR), to distinguish it from reactivation or reinfection.

This data demonstrated a statistically significant association between women who were exposed to abortion two times and positive anti-CMV IgM antibodies (PR = 5.0, 95% CI: 1.13–21.97; *p* = 0.042). Previous studies reported no significant associations [2, 7, 15, 18]. All positive CMV IgM recurrent cases in the present study had a period of marriage of less than 7 years and became pregnant less than three times (Table 3).

## Limitations

This study has several limitations that should be acknowledged. First, its cross-sectional design precludes establishing temporal or causal relationships between CMV seropositivity and spontaneous abortion. Second, the absence of a control group limits comparison of CMV seroprevalence between women with and without spontaneous abortion. Third, the relatively small sample size may have reduced the statistical power and limited the generalizability of the findings. Fourth, univariate analysis was performed, and multivariable analysis was not conducted due to limited sample size, particularly for IgM-positive cases (*n* = 8), which precluded stable multivariable modeling. Therefore, the potential confounding factors were not adjusted for reported associations.

In addition, due to financial and logistical constraints, confirmatory molecular testing using PCR and CMV IgG avidity testing was not performed. Consequently, it was not possible to distinguish primary CMV infection from viral reactivation or reinfection. Furthermore, other infectious causes of adverse pregnancy outcomes, including TORCH pathogens such as Toxoplasma gondii, rubella virus, and herpes simplex virus, were not investigated in the present study.

Prevalence ratios were used in this cross-sectional study, as they are more appropriate than odds ratios for measuring associations in prevalence studies [27]. While odds ratios are commonly reported in case-control studies, they tend to overestimate associations when the outcome is common (> 10%), as is the case with CMV seroprevalence in our population [50]. However, as with all prevalence ratios, these measures describe associations and do not establish causality.

## Conclusions

The seroprevalence of anti-CMV IgG antibodies was substantially higher than that of anti-CMV IgM antibodies in Lahj Governorate, Yemen. In this study, anti-CMV IgM seropositivity was significantly associated with having experienced two previous spontaneous abortions. Although a history of blood transfusion was associated with an increased prevalence of anti-CMV IgG seropositivity, this association did not reach statistical significance. Larger prospective studies incorporating PCR, CMV IgG avidity testing, appropriate control groups, larger sample sizes, and multivariable analyses are required to clarify the relationship between CMV infection and spontaneous abortion and to better understand its role in adverse pregnancy outcomes.

## Data Availability

All data was presented in the manuscript, no further data available and it will be available at any time.

## Abbreviations

95% CI: 95% confidence interval
AIDS: Acquired immunodeficiency syndrome
CMV: Cytomegalovirus
ECLIA: Electrochemiluminescence immunoassay
IgG: Immunoglobulin G
IgM: Immunoglobulin M
IQR: Interquartile range
PCR: Polymerase chain reaction
PR: Prevalence ratios
TORCH: *Toxoplasma gondii*, rubella virus, cytomegalovirus and herpes simplex virus
UD: Undefined
